# Does a school-based intervention to engage parents change opportunity for handwashing with soap at home? Practical experience from the *Mikono Safi* trial in Northwestern Tanzania

**DOI:** 10.1371/journal.pntd.0010438

**Published:** 2022-06-06

**Authors:** Yovitha Sedekia, Saidi Kapiga, Onike Mcharo, Kenneth Makata, Belen Torondel, Robert Dreibelbis, Elialilia Okello

**Affiliations:** 1 Mwanza Intervention Trials Unit, Mwanza, Tanzania; 2 Department of Infectious Disease Epidemiology, London School of Hygiene and Tropical Medicine [LSHTM], London, United Kingdom; 3 Department of Disease Control, London School of Hygiene and Tropical Medicine [LSHTM], London, United Kingdom; University of Glasgow School of Life Sciences, UNITED KINGDOM

## Abstract

**Background:**

School-based de-worming is advocated as a strategy for reducing the burden of soil-transmitted helminth (STH) infections among children. However, re-infection tends to occur rapidly, suggesting that comprehensive water, sanitation, and hygiene (WASH) improvements may be needed to prevent this. We qualitatively assessed the influence of parental engagement activities on parents’ motivation to improve WASH infrastructure and hygiene practices at home in the context of a school-based de-worming programme.

**Methodology:**

We conducted a longitudinal qualitative study nested within the *Mikono Safi* trial, designed to assess the effect of a WASH intervention on STH infection prevalence in children. Meetings were organized for parents/guardians at schools where they were given information about STH infection, the role of WASH in STH infection prevention, and actionable steps they could take at home. During the meetings, parents/guardians received information about their own child’s STH infection status. Twenty purposively selected households were visited and interviewed 3 times over a period of about 8-months. We employed thematic analysis; findings are reported following the Capability-Opportunity-Motivation and Behaviour (COM-B) framework.

**Principal findings:**

The engagement strategy improved parents’/guardians’ knowledge and skills about handwashing with soap and its benefits. Parents/guardians reported that the sessions had motivated them to improve WASH infrastructure at home. Of 20 households included in this study, 17 renovated or built new latrines and 18 installed handwashing facilities. However, only 8 households established and maintained handwashing stations with both soap and water at 8 months.

**Conclusions:**

The engagement of parents/guardians in a school-based WASH education intervention as part of the *Mikono Safi* trial resulted in increased knowledge and motivation about handwashing and sanitation. This led to improvements in sanitation facilities and handwashing opportunities at home. However, long-term success in provision of water and soap was limited, indicating that sustained engagement may be required to encourage households to ensure these materials are consistently available at home.

## Author summary

School-based de-worming is used to reduce soil-transmitted helminth (STH) infections among children. However, re-infection occurs rapidly. The *Mikono Safi* trial focused on enabling and motivating children to wash their hands at key times, with the aim to reduce the proportion of children with STH infection. Parents/guardians invited to participate in parental engagement activities at school as part of the trial, were given information on how STHs can be avoided through improved handwashing practices at school and at home. To assess the effect on parental engagement activities, we conducted 20 qualitative interviews with parents/guardians and WASH observation in 20 households. We found that the engagement activities were successful in increasing parents’/guardians’ concern about STH infection in their children, which in turn led them to improve handwashing opportunities and practices in the home. However, not all households had improved handwashing facilities at home or ensured that water and soap was available.

## Introduction

Soil-transmitted helminth (STH) infections are the most common neglected tropical disease worldwide with up to 1 billion school-aged children living in endemic areas of at least one STH [1,2]. To address the large burden of morbidity and associated developmental consequences for children, school-based de-worming campaigns are recommended by the World Health Organisation (WHO) as the most cost-effective strategy to address STH infections, allowing relatively easy access to children in schools in poor rural areas [3–6].

Although STH infections are easily treated with albendazole and mebendazole, re-infection occurs quickly [7,8]. There are a number of factors that drive the prevalence of STH infection among school children. For example, the lack of sanitary facilities at home is three times more likely to increase a child’s risk of acquiring hookworm infections [9–11]. A systematic review on the impact of sanitation found that improved sanitation at home was associated with 27% lower odds of *A*. *lumbricoides* infection, 20% lower odds of *T*. *trichiura* and 35% lower odds of hookworm infection [12]. Access to clean water is important to prevent the spread of infectious diseases [13], including intestinal helminths [14–17].

According to the updated atlas of human helminth infections [18], STH infections are highly prevalent in most regions of Tanzania, with regions along Lake Victoria, including Mwanza, Kagera and Mara, having the highest burden of the infections. The prevalence of *Ascaris lumbricoides* and *Trichuris trichiura* ranges from 18% to more than 50% in Kagera region [18,19], indicating the need for urgent interventions that could reduce the burden of STH in school-aged children.

Multiple studies have assessed the effect of combining school-based de-worming with school-based WASH improvements and school-based health education targeting children [20–22]. However, a review of WASH interventions shows conflicting results on relationship of these interventions with STH infection [23]. Few trials have tested how parental engagement activities as part of a comprehensive WASH intervention can motivate parents and guardians to improve their sanitation and handwashing situation at home [24]. This paper reports results of a qualitative study conducted to assess the influence of school-based parental engagement activities regarding STH infection on WASH conditions and practices at home.

## Methods

### Ethics statement

The *Mikono Safi* trial received ethical approval from the review boards at the London School of Hygiene and Tropical Medicine (LSHTM) (**Ref: 11868**) and the National Institute for Medical Research (NIMR) in Tanzania (**Ref: NIMR/HQ/R.8a/vol.IX/2497**). This longitudinal qualitative study, received additional ethical clearance number (**Ref: 14539**) from LSHTM and (**Ref: NIMR/HQ/R.8a/IX 2665**) from NIMR.

Prior to the data collection, participants received information sheets explaining the study aim and procedures. Participation was voluntary and participants were informed of their rights to refuse to participate or withdraw from the study at any point should they wished to do so without a fear of being penalised or denied their health and social rights in the community. Parents provided written, informed consent to participate in the in-depth interviews and for their latrines and handwashing facilities to be observed up to three times in a year. Personal identification data in the transcripts and reports were removed to maintain the respondents’ anonymity and confidentiality. There were no payments for participating in the study.

### Study setting

This qualitative research was nested in the *Mikono Safi* trial which was conducted in 16 schools (8 intervention and 8 control schools) in Kagera region in Northwestern Tanzania [24,25]. Kagera region is predominantly rural and is comprised of 8 districts with an estimated population of over 2.7 million [26]. The region is situated on the western shore of Lake Victoria, neighbouring Rwanda and Burundi on the west and Uganda to the north. Its economy depends mainly on agriculture. All major villages of the region have primary schools to class 7 and about 90% of children attend the school for at least some years [27]. Primary schools may comprise up to 1500 children aged 6–14 years, with an average of 80 students per classroom (range 65–93 students) in 2019 [28]. Kagera region was selected because of its high prevalence of STH species (*Ascaris lumbricoides* and *Trichuris trichiura* infections), predominantly transmitted through oral ingestion of worm eggs [18,19,25].

Details of the *Mikono Safi* trial have been published elsewhere [24,25]. Briefly, the trial aimed to determine whether the effect of routine de-worming on the prevalence of *Ascaris lumbricoides* and *Trichuris trichiura* infections among school aged children can be sustained when combined with a behaviour change intervention promoting handwashing with water and soap [24,25]. The intervention involved classroom-based hygiene promotion and structural change within the school environment. The classroom-based hygiene promotion comprised of three sessions of about 60–80 minutes taught at school by three trained teachers over the course of one year. The sessions were delivered to pupils aged between 6 and 13 years in standard 1 to 6 of primary school. Games, posters and sets of comic-type pictures were used to deliver the lessons that focused on important emotional drivers that would motivate the school children to adopt better hygiene. The structural change within the school environment included low-cost, replicable infrastructure modifications on school grounds, including installation of handwashing facilities near school latrines, construction of footpaths connecting the handwashing station to the toilet block, and nudges to prompt hand washing behaviour [24].

The intervention also included a targeted strategy for engaging parents. Stool specimens were collected from all students from the intervention schools for STH infection screening prior to the trial implementation. Parents/guardians were then invited to attend a meeting at their child’s school where general information about intestinal worm infections and handwashing was provided. Stool test results of their children were provided in a closed envelop to each parent along with a discussion of what each individualised child’s results meant. At the end of the session, facilitators discussed possible changes that parents can make at home to prevent infection or re-infection [24].

### Study design

The Capability-Opportunity-Motivation and Behaviour (COM-B) framework [29] was used to guide our study design, data collection and analysis. In the COM-B, the “capability”, “opportunity” and “motivation” are the essential determinants of a “behaviour” outcome. In our study, capability included handwashing skills and knowledge; opportunity included parent’s ability to install handwashing facilities and/or improve household latrines by either putting a door or curtain to improve privacy, reducing the size of squatting hole to improve safety, roofing, building new toilet, and consistently provide soap and water for handwashing at key points. Motivation included emotional and motivational drivers to changing households’ latrines and handwashing facilities. The behavioural outcome of interest included improving latrines and installing and maintaining handwashing facilities, including having both water and soap available for washing hands.

The study was conducted in the catchment area of two primary schools receiving the *Mikono Safi* intervention in Muleba district, Kagera region. Data were collected at participating households using direct spot check of household handwashing and sanitation facilities and in-depth interviews (IDIs) with parents or guardians of school children. A total of three rounds of data collection for each participating household were conducted between April and November 2018. Baseline data were collected before the parental engagement activities and two additional rounds at four and eight months following the sessions.

### Study population, sampling and sample size

Two schools were purposively selected for participation in this nested qualitative study. A total of 20 parents/guardians (10 per school) from 20 different households were enrolled. In each of the two schools, we purposively selected five parents of children with moderate to high intensity STH infections and five parents of children with no infections at baseline. The WHO STH infection intensity category of helminths infections > 5,000 eggs/gram of faeces for *Ascaris lumbricoides* or > 1,000 eggs/gram of faeces for *Trichuris trichiura* [30] was used as a definition of high infection for the qualitative study. Details on stool sample collection, processing and analysis are provided elsewhere [24]. The sampling list for this qualitative study was developed with the help of the trial coordinator who had access to worm infection results. In each of the two schools we developed a list of 14 students (7 medium to high infection; 7 no infection) but the list did not indicate infection status. The school head teacher, who was blinded to the infection status of the children, helped to select 5 students from each sub category.

### Data collection and quality control

#### Household-data collection

Selected households were involved in three rounds of data collection. The first round (baseline) data collection was conducted two weeks before parents were invited to attend engagement activities at the school and receive laboratory results for their children worm tests. The data collection team did not disclose information on worm infection to the parents/guardians during baseline data collection. The second round of data collection was conducted four months after parental engagement activities to assess if or how parents had made improvements to sanitation and handwashing facilities at home. The third and final round was conducted approximately four months after the second round to assess sustainability of changes that had been made by households to improve opportunity for handwashing and hygienic use of latrine. The interview invitation was sent to the child’s parents/guardians and we aimed to interview one parent/guardian available at the household on the day of the visit. In case both parents/guardians were available, they sat in the interview and responded to the interview questions together. During the subsequent visits, deliberate efforts were made to ensure that the same parent(s)/guardian(s) were interviewed (Table 1). The following activities were conducted at each round of data collection:

*i. Household spot checks*:

A semi-structured spot check tool was completed in each household. The tool captured information on current state of household latrines and latrine quality—including privacy and whether the toilet was safe to be used by young children—and the availability and location of handwashing material.

*ii. In-depth interviews with parents or guardians of school children*:

A semi-structured topic guide with questions on perceived knowledge about handwashing and helminths infection (capability); available latrines and handwashing facilities at home (opportunity); and influence of the parental engagement activities on motivation for handwashing at home was used. The guide was developed in English, translated into Swahili, pilot-tested and refined before each round of data collection.

The interviews were conducted in person at the respondents’ homes in a place that provided privacy and at a time convenient to the respondent. Two experienced research assistants received three days of training followed up by two days of piloting the interview guides. All interviews were recorded with a digital device. The average duration was 27 minutes. After each interview, the team reviewed the interview and field notes for completeness of information and identified any emergent findings that needed to be explored further in the next interview.

**Table 1:** Study participants and their school children’s characteristics.

	Parents of children with STHs			Parents of children without STHs		
**Round of data collection**	1^st^ Rounds (Baseline) (April 2018)	2^nd^ Round (August 2018)	3^rd^ Round (November 2018)	1^st^ Rounds (Baseline) (April 2018)	2^nd^ Round (August 2018)	3^rd^ Round (November 2018)
**Age range**	27–89	27–92	27–92	32–67	26–78	26–57
**Mean age**	38	46	47	46	45	39
**Relationship**						
Male parents	4	4	4	2	2	3
Female parents	5	5	5	7	7	7
Guardian(s)	1	1	1	1	1	0
**Same parents/guardians interviewed during 2**^**nd**^ **and 3**^**rd**^ **rounds**						
Yes	N/A	8 (from baseline)	10 (from 2^nd^ round)	N/A	6 (from baseline)	9 (5 from baseline & 4 from 2^nd^ round)
No	N/A	2	0	N/A	4	1
**Level of Education**						
Formal education (completed primary and above)	5	5	5	8	8	8
No formal education	4	4	4	1	2	2
No information recorded	1	1	1	1	0	0
**Occupation**						
Peasants	10	10	10	8	8	8
Teachers	0	0	0	1	1	1
Business owners	0	0	0	1	1	1
**Contact with Intervention**						
A parent/guardian or family members attended parental engagement activities	0	10	10	0	10	10

### Data management and analysis

All recorded in-depth interviews were transferred after each interview to the Mwanza Intervention Trials Unit (MITU) computers and server which are password-protected. The audio recordings were transcribed verbatim in Swahili. The Swahili transcripts were managed and coded using NVivo 12 software. Initial analysis happened concurrently with data collection and was an iterative process with findings from each round of data collection and analysis informing the subsequent data collection and analysis. For example, those whose household had no latrines or handwashing facility during the first or second round of data collection were asked in the second and third round, respectively, on whether they constructed latrines or installed handwashing facilities. Those who had toilets that needed improvements were asked during the second or third round about the improvements made, etc. We employed thematic analysis that involved i) familiarization of data through re-listening to audio and/or re-reading of transcripts and field notes; ii) initial coding guided by the COM-B Framework [29,31]; iii) searching for themes; iv) reviewing themes; v) defining and naming themes; vi) and writing up the report [32,33]. Initial key themes were identified by the first author (YS) and discussed with the last author (EO). We entered spot check data into an excel sheet and compared household condition of toilets, handwashing facility and availability of soap across the three rounds of data collection.

## Results

A total of 60 in-depth interviews were conducted during the three rounds of data collection. Seventeen respondents were the parents of the children and three were guardians (two grandparents and one uncle). The majority of them had completed primary school and were subsistence farmers. Ages ranged between 26–92 years with mean age of 44 (Table 1).

### Parental engagement sessions: Reach and engagement

#### Parents/guardians prior knowledge about the Mikono Safi intervention

During the first round of data collection, most parents/guardians reported they had received information from their children about the *Mikono Safi* activities taking place in the schools. Additionally, they had heard about stool sample collection among school children, school toilet renovations, handwashing station installations and hygiene educational sessions provided to children at school.

*“****R*:** *Eeee*, *recently the child mentioned something about people helping to renovate the [school] toilet*. *The child said a lot of things about washing hands with soap but I really don’t remember the details”* ~**Participant 4, no infection group, school 1, round one**

By the second round of data collection, all parents/guardians reported that they or a representative of their household had attended the parental engagement activities at the school attended by their children. During the session, parents/guardians reported they had received information about STH infection, role of handwashing in STH transmission, stool test results and their meaning. They also participated in a discussion about the potential changes they could do at home to prevent infection or re-infection.

*“****R*:** *Yes*, *my household was invited for the meeting at school…the meeting was in May if I can remember correctly… I am the one who attended…I was given the child’s worm test results… [the child] had worm [infection]…”* **~Participant 9, high infection group, school 2, round two**

#### Parents’ reactions to the STH test results

Parents/guardians reported various reactions after receiving their child’s STH test results. Parents/guardians whose children had no worm infection reported being happy and pleased by the results. Most parents/guardians whose children tested positive raised concerns about their children’s health, while some were surprised to learn that their children had so many worm eggs in their bodies.

*“****R*:** *I felt sad*, *I was so surprised…other parents’ children have zero*, *[but] mine have two hundred plus*!*…I came back home and told their mom “do this and this*, *let’s try our best”…*, *but they came back to de-worm them and later on we received a second round of results”* **Participant 2, high infection group, school 1, round two**

### Parental engagement influence on capability for handwashing

During the first round of data collection, most parents/guardians reported that their children had some knowledge about handwashing, particularly on when to wash hands but had not noticed any recent changes in the children’s handwashing behaviours. The children washed hands in the same way they used to, often before and after food time and after using a toilet.

*“****R*:** *for sure*, *I haven’t noticed any changes in handwashing behaviour*, *the child washes hands as we are normally advised*. *She/he washes before eating food*, *after eating food and after using the toilet…”* **~Participant 6, high infection group, school 2, round one**

During the second round of data collection, the majority of parents/guardians in both the high and no infection groups reported improved hand washing knowledge and skills in their children. The children were washing their hands properly using soap and running water and that children had developed a handwashing habit at key points, particularly after using the toilet. Parents/guardians reported that children often asked or looked for water or soap before handwashing.

*“****R*:** *…as I told you earlier*, *nowadays children are keen to wash hands unlike when we had yet received the handwashing training…*.*nowadays it is a must to wash hands after using the toilet*, *something that was not usually practiced before…*.*during food time they used to wash their hands in a rush but nowadays they scrub to ensure that they remove all the dirt…*.*at meal time they wash hands with running water****”* ~Participant 3, high infection group, school 1, round two**

During the third round of data collection, parents/guardians reported that the children handwashing behaviour after using the toilet was sustained but that how well children washed hands varied. Sometimes they washed thoroughly and other times they did not wash as thoroughly as they had been taught.

*“****R*:**…*what makes me believe that they understand more on how to wash hands it’s because I see them washing their hands*, *I observe them*. *But honestly speaking they don’t wash their hands thoroughly as they were taught at school*… .*here at home*, *they wash their hands without scrubbing*… .*that is difficult for children to adhere to*. *They scrub their hands once in a while but often time they just wash the way they were used to do*, *not as they were taught to do*… .*you cannot stay with children all the time*, *so when you are not around*, *they do not bother much about handwashing”* **~Participant 2, no infection group, school 1, round three**

Some parents/guardians mentioned that they were unable to observe how children washed hands due to competing priorities. The only times they were confident that the children washed their hands properly were mealtimes when the parents could observe handwashing.

*“R*: *Immediately after the training I used to closely observe how they washed their hands that’s why I said that they understand well how to wash hands*, *but nowadays we are so busy with farms…every day we wake up in the morning and it becomes difficult to know whether they continue washing their hands properly or whether they have already forgotten…but during meals time I see them wash their hands as they were taught to do…”* **~Participant 8, no infection group, school 2, round three**

### Parental engagement influence on the opportunity for sanitation and hygiene at home

#### Latrines

**Fig 1:**
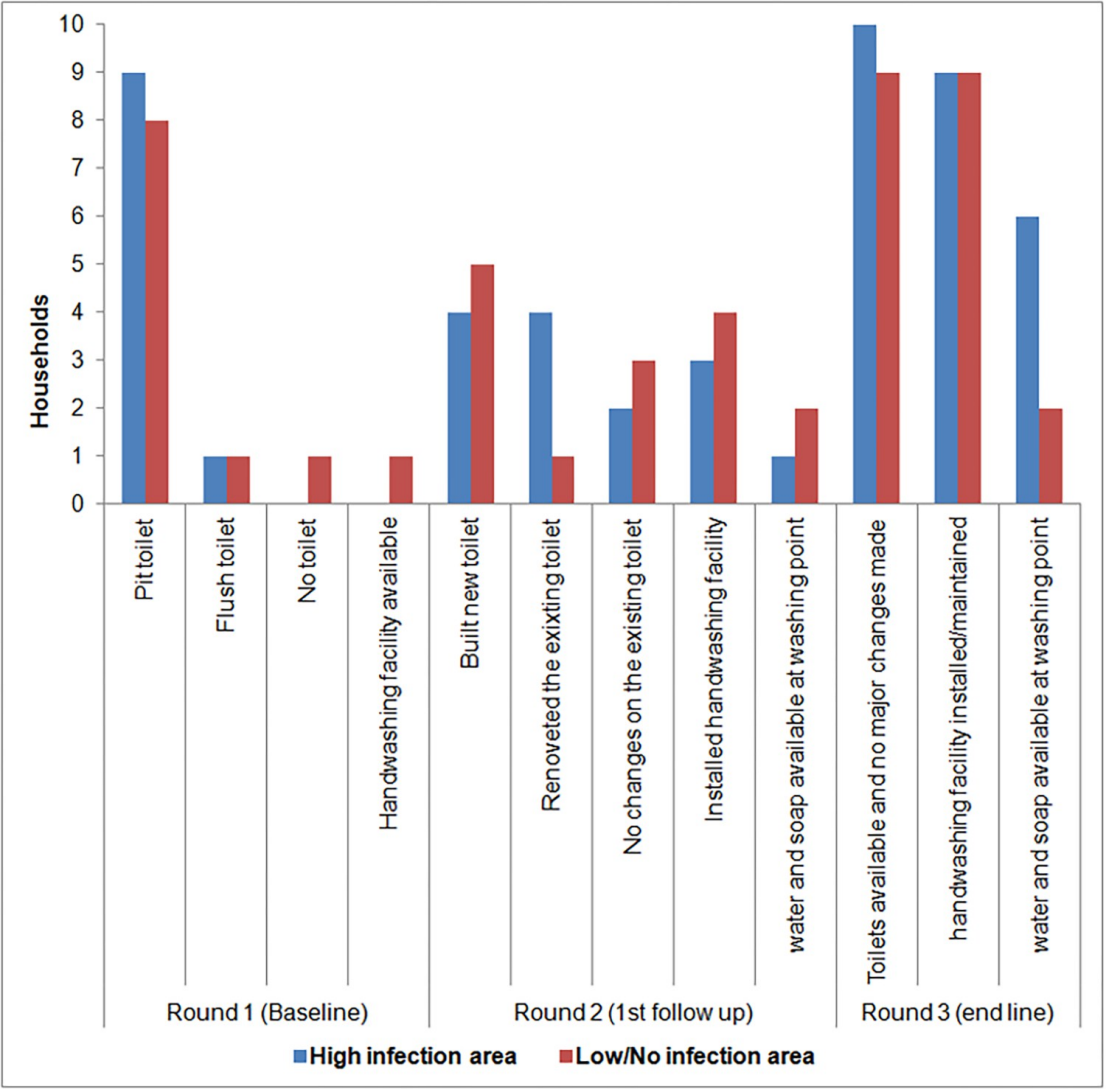
State of household sanitation (toilets and handwashing facilities at baseline, at 4 and 8 months).

Based on direct observations during the first round of data collection, almost all households had latrines within the compound. Of these, 17 (9 from high intensity infection group and 8 from no infection group) were open pit latrines constructed from locally available materials while two (1 from each group) were flush latrines. Most of walls of the open pit latrines were made of dry wood, dry banana leaves, or mud and wattle or old pieces of iron sheets, while the floor/slab was made of dry wood or timber. Fig 1 below shows changes made on toilets after the parental engagement activities

Of the households with latrines, 12 had latrines that were unsafe for children use due to factor such as inappropriate distance between the foot rest and squatting platform, the diameter of squatting hole, unsafe distance between the wood slats of the floor, or distance from the household to the toilet. Although most latrines had signs of recent use, 13 of the open pit latrines lacked privacy because they either had no door, the walls were too short to cover someone inside the toilet, or the walls had gaping holes (Fig 1).

By the second round of data collection, 13 out of 17 households with open pit latrines had made some modification (8 from high intensity infection group and 6 from no infection group). The modifications ranged from adding a simple door to improve privacy to adding wood to the floor to improve safety for children. Nine households had constructed new latrines (4 from high intensity infection group and 5 from no infection group) (Fig 1).

The observations were corroborated during the in-depth interviews with parents/ guardians. During the first round of data collection, no recent renovations or changes on latrines were reported. They also reported that they normally build a new traditional pit latrine rather than repairing an old and/or unstable toilet. The male head of the household was often responsible for building latrines in the households.

“***R*:** *It is not easy to renovate our traditional latrines because their inside walls are not made of stones…*. *Whenever the toilet starts to wear out the soil walls slide and there is no other way you can renovate it……you renovate it by digging a new toilet…*.*you knock down the current one*, *cover it with soil and prepare another one…*.*”* **~Participant 9, no infection group, school 2, round one**

During the second round of data collection, the majority of respondents reported they had built new toilet using labour and material available within the household.

*“****R*:** *As I have told you*, *when you visited us there was no toilet here at home*, *there was dilapidated toilet but after the training I came back and mobilised people in my household and we dug another toilet…I covered it using dry timbers*, *I put soil [on top of the timbers] and grasses on the roof as they had told us to do*, *but we didn’t put the pipe at the back…”* **~Participant 7, high infection group, school 2, round two**

By the third round of data collection only one household from no STHs infection group had not completed building a new toilet. The respondents reported financial constraints as the primary barrier to new latrine construction, and they reported using a neighbours’ toilet (Fig 1). Most parents/guardians reported that their latrines were still in good condition and they had not made any major changes since the second round of data collection.

#### Ensuring latrines are clean

It was reported that female parents/guardians were responsible for cleaning the latrines. It was mentioned that cleaning was done daily using locally made sweeping materials. Respondents reported pouring ashes on the floor around squatting hole as both a disinfectant and an air freshener. It was also mentioned that cleaning supplies were kept in the toilet to ensure that they are always available.

#### Handwashing facilities

In our observation during the first round of data collection, only one household (with a flush toilet) had a handwashing facility and piped water within the compound. During the second round of data collection, seven households had built a handwashing facility but only 3 had water and soap at these handwashing facilities at the time of data collection. During the third round of data collection, almost all households had handwashing facilities. However, the majority of households had water but no soap at the handwashing facility. Only 8 households had both water and soap. One household did not install handwashing facility and our impressions during data collection was that the household head was not interested in improving sanitation and hygiene for his household (Fig 1).

During the third round of data collection, most parents/guardians reported they had not made any major changes on their handwashing facilities. However, some parents/guardians reported they had purchased more water storage containers and other equipment that could serve as a handwashing facility.

*“****R*:** *Regarding handwashing facilities*, *I have not changed anything except I added an additional water container because the one we had initially was not enough*. *These days Children are very active in washing hands so the water would not last*. *So I decided to have an additional water container*. *I added the water container because the water was not enough; otherwise*, *I have not made major changes*. **~Participant 7, high infection group, school 2, round three**

#### Ensuring water is available

Most parents/guardians reported consistent availability of water in their households. During all rounds of data collection, parents/guardians reported that they were able to collect water directly from multiple sources within their community; a few parents/guardians reporting buying from water vendors especially during dry season.

### Parental engagement and impacts on motivation for the changes on latrines and handwashing facilities

Parents/guardians reported multiple motivations for changing their household hygiene and sanitation after the parent engagement session. Parents/guardians from both groups reported that the parental engagement sessions helped them understand not only the sources of STH infection but that changes that they could make to reduce the risk of STH infection were possible using locally available materials.

*“****R*:** *…when we attended the parental engagement meeting*, *we were taught about the improvement that we could make and we made them*. *When we were taught about worms*, *we decided to improve the toilet for we were told worm infection was dangerous to children’s health…”* ~**Participant 3, high infection group, school 1, round two**

Parents from the high STH group mentioned embarrassment of having STH positive results and fear of consequence STH may have on the health of a child as key motivation to improve WASH facilities

***“R*:** *The motivation to change the toilet was when my child was found to have worm infection…”* ~**Participant 5, high infection group, school 1, round two**

A few respondents in both high and no infection groups reported that embarrassment about the poor state of their latrines at baseline partly motivated them to change their latrines or handwashing facilities.

## Discussion

This study assessed the influence of parental engagement activities on WASH conditions and practices at home in the context of school-based multi-component intervention. Our study findings demonstrate that parental engagement activities positively influenced access to handwashing facilities and clean latrines at home. However, the *Mikono Safi* intervention was unable to address persistent barriers to effective sanitation and hygiene in the home.

The targeted parental engagement activities increased not only parents’ knowledge about transmission and prevention of helminths infections but also the awareness that changes to latrines and handwashing facilities can be made using locally available materials. Lack of knowledge about how to change latrine or handwashing facility using locally available materials can hinder such changes in homes. A qualitative study in Kenya found that participants in three-quarters of the focus group discussions reported high cost of latrines’ building materials to be one of the reasons for low latrine coverage in their community [34]. Although all the changes were done using basic and locally available materials to renovate toilets and install handwashing facilities, the demonstrated changes in sanitation and hygiene infrastructure at home show that people can be receptive to a hygiene-related intervention when solutions to overcome perceptions around cost and resources are provided [35,36].

Parents have a great influence over their children’s health promotion behaviours [37,38]. With growing evidence about the effect of combined interventions on preventing the spread of infectious diseases in general and intestinal helminths in particular [20–22], the parental engagement model tested in this study is a valuable addition to sanitation and handwashing interventions targeting school children. Engaging and involving parents in a school-based WASH program creates a synergy for the parents/guardians to improve WASH infrastructure at home and could increase intervention impact beyond the school setting.

We found that the knowledge on STH infection and prevention as well as skills on how to make structural changes at home provided during the parental engagement sessions motivated parents to make changes. In addition, receiving positive STH infection test results evoked concerns about their children’s health and prompted parents/guardians to improve their sanitation situation and handwashing facilities at home. The nurture emotion has been reported in other settings to be a useful motivation in triggering changes in latrine condition and handwashing facilities [36,39–41]. This implies that targeted parental engagement activities may be an important additional platform for school-based interventions targeting to influence motivation for improving sanitation and hygiene at home. Rather than abstract health information, STH test results gave parents and guardians specific information about their own child’s health. Providing tailored, customized information about water quality was associated with reduced levels of household water contamination in a trial in India [42], and efforts to provide specific information about environmental health risks warrant further exploration.

While most parents/guardians improved latrine and hand washing facilities in the households, only about 40% established and maintained a handwashing station with both soap and water in the homes. In some households, economic/financial barriers may have contributed to general lack of soap in the household or rationing soap for critical uses. Households with handwashing facilities are roughly twice as likely to wash their hands with soap as compared to their counterparts who lacked a facility [43]. In our study, parental engagement sessions influenced the opportunity for handwashing at home across all groups installing handwashing facilities. Parents perceived handwashing with soap among children to have improved, particularly washing hands with soap after using the toilet. Availability of improved latrines and functional handwashing facilities at home is one thing but sustaining availability of water and soap at the handwashing point is something else. In our study, despite the reported changes in knowledge and parental engagement activities, parents across groups were unable to consistently provide soap for washing hands. This is not a unique finding to our study [36] and it confirms that while provision of soap and water next to the latrine encourages good hygiene behaviours, sustaining such behaviours within everyday settings might be challenging. Often time households have soap in their homes but not at the handwashing points [36].

The extent to which parental engagement activities and changes in the household translated to increased handwashing among school going children requires further investigation. Some studies found that amount of time children spend with parents affected children’s engagement in handwashing practices [37] and major barriers to washing hands among children were forgetting to wash hands (63%) and being in hurry (24%) [44]. In our study, parents across groups closely supervised and observed their children in the early days after the parental engagement sessions but as days progressed, they were occupied with other daily activities and had less time to spend with their children to observe their handwashing practices. Therefore, future research should systematically examine the effect of school-based handwashing with soap interventions on capability and behaviour in the homes.

### Limitations

Our study has the following potential limitations: first, this was a small sample of parents/guardians in 20 households, drawn from two schools out of the eight *Mikono Safi* intervention schools. Results may not be generalisable. However, the parents were purposively selected to include those with children with high intensity of infections and those without infections. Secondly, the observed changes in latrines and handwashing facilities could be due to Hawthorne effect caused by the parent’s knowledge that research team would visit the household repeatedly. As indicated in the ethical considerations, parents were asked for signed informed consent prior to the data collection to be interviewed and for direct spot check of the household latrines and handwashing facilities up to three times in a year. Participants were aware that their latrines and handwashing facilities would be directly observed during each visit. However, due to a longitudinal nature of the study, there is a possibility that the Hawthorne effect was minimized. In addition, the two subsequent household visits were unannounced, so the interviewed parents would not know the exact time point of the following visit. Also, the interval between the visits was spaced approximately 4 months apart; an interval in which a behaviour change is unlikely to be sustained by the Hawthorne effect alone. Social desirability bias is introduced when study participants respond to self-reported items in a way they think the interviewer wants to hear, instead of providing accurate/truthful responses [45]. Despite intensive training of research assistants and supervision, social desirability bias cannot be eliminated particularly with regards to reporting perceived motivation for changing latrines and handwashing facility conditions at home. Through the combination of interviews and spot check data and the longitudinal nature of the study, we were able to minimize the desirability bias. In addition, data analyses were carried out in the Swahili language by a fluent English and Swahili speaker to preserve the original meaning of the responses to the extent possible. Finally, our research was conducted among participants with limited resources and some participants, especially those with children from the high infection group, reported lack of soap due to financial constraints. Therefore, while a small amount of soap is sufficient for hand washing, household that could not afford soap for general household use, were unlikely to afford soap for handwashing. However, even those who reported having soaps within their households they did not consistently put the soap at the hand washing points. In addition, with regards to improving latrines and handwashing facilities, the reported changes though significant and important changes, they were basic and used locally available materials within their households. However, it was clear from our data that large scale infrastructural improvement may be out of reach for many low-income households. Additionally, the incremental infrastructure improvements may not be sufficient to interrupt transmission [46]. However, our finding shows the need to make behaviour change part of a larger comprehensive package for larger environmental health interventions.

## Conclusion

This study explored how a targeted parental engagement session on STH infection as part of a school-based WASH behaviour change intervention influenced targeted emotional motiviations of household behaviours. The emotional motivations resulted in infrastructural changes at home. However, long-term success in provision of water and soap, which requires repeated time and financial investments, was limited. Additional intervention strategies may be required to encourage households to help ensure water and soap are consistently available.
